# Maternal time investment in caregiving activities to promote early childhood development: evidence from rural India

**DOI:** 10.3389/fped.2023.1120253

**Published:** 2023-07-07

**Authors:** Neha Batura, Reetabrata Roy, Sarmad Aziz, Kamalkant Sharma, Divya Kumar, Deepali Verma, Ana Correa Ossa, Paula Spinola, Seyi Soremekun, Siham Sikander, Shamsa Zafar, Gauri Divan, Zelee Hill, Bilal Iqbal Avan, Atif Rahman, Betty Kirkwood, Jolene Skordis

**Affiliations:** ^1^ Institute for Global Health, University College London, London, United Kingdom; ^2^ Department of Population Health, London School of Hygiene and Tropical Medicine, London, United Kingdom; ^3^ Child Development Group, Sangath, New Delhi, India; ^4^ Department of Anthropology, University College London, United Kingdom; ^5^ Department of Infection Biology, London School of Hygiene and Tropical Medicine, London, United Kingdom; ^6^ Department of Primary Care and Mental Health, University of Liverpool, Liverpool, United Kingdom; ^7^ Global Institute of Human Development, Shifa Tameer-e-Millat University, Islamabad, Pakistan; ^8^ Fazaia Medical College, Air University, Islamabad, Pakistan

**Keywords:** time use, child health, early childhood development, India, time allocation

## Abstract

**Introduction:**

Intervention strategies that seek to improve early childhood development outcomes are often targeted at the primary caregivers of children, usually mothers. The interventions require mothers to assimilate new information and then act upon it by allocating sufficient physical resources and time to adopt and perform development promoting behaviours. However, women face many competing demands on their resources and time, returning to familiar habits and behaviours. In this study, we explore mothers' allocation of time for caregiving activities for children under the age of 2, nested within a cluster randomised controlled trial of a nutrition and care for development intervention in rural Haryana, India.

**Methods:**

We collected quantitative maternal time use data at two time points in rural Haryana, India, using a bespoke survey instrument. Data were collected from 704 mothers when their child was 12 months old, and 603 mothers when their child was 18 months old. We tested for significant differences in time spent by mothers on different activities when children are 12 months of age vs. 18 months of age between arms as well as over time, using linear regression. As these data were collected within a randomised controlled trial, we adjusted for clusters using random effects when testing for significant differences between the two time points.

**Results:**

At both time points, no statistically significant difference in maternal time use was found between arms. On average, mothers spent most of their waking time on household chores (over 6 h and 30 min) at both time points. When children were aged 12 months, approximately three and a half hours were spent on childcare activities for children under the age of 2 years. When children were 18 months old, mothers spent more time on income generating activities (30 min) than when the children were 12 years old, and on leisure (approximately 4 h and 30 min). When children were 18 months old, less time was spent on feeding/breastfeeding children (30 min less) and playing with children (15 min). However, mothers spent more time talking or reading to children at 18 months than at 12 months.

**Conclusion:**

We find that within a relatively short period of time in early childhood, maternal (or caregiver) time use can change, with time allocation being diverted away from childcare activities to others. This suggests that changing maternal time allocation in resource poor households may be quite challenging, and not allow the uptake of new and/or optimal behaviours.

## 1. Introduction

The first five years of life lay the foundation for the development of human capital (1). This period is critical for the development of children's emotional, physical, and intellectual well-being (2, 3). Investments in children's health and development during this period can play a very important role in the accumulation of human capital in the short-term and throughout the life course (4). However, exposure to physical risks factors such as poor maternal nutrition, low-birthweight and infectious diseases; and psycho-social risks such as maternal depression, exposure to violence and lack of stimulation or nurturing care can prevent children reaching their full potential, especially in resource constrained settings (5–7).

In 2016 it was estimated that in low- and middle-income countries (LMICs), 250 million children under the age of five years were at risk of impaired cognitive and social-emotional development (8). Several strategies have attempted to improve early childhood development (ECD) outcomes (9–14). Typically, ECD programmes tend to be targeted at the primary caregiver(s) of children, and delivered through home visits, community groups, clinic or facility-based services or media campaigns. They promote optimum childcare practices, including infant and young child feeding, nurturing care through more engaged and responsive caregiver-child interactions, creating appropriate home environments and stimulation through play (3, 14–17). These programmes could lead to higher parental investment in their children's health and development in terms of physical resources and time (4). They could also directly impact ECD outcomes, either by enhancing the effectiveness of existing levels of parental inputs or acting as a new input for improved ECD (4).

For ECD programmes to improve outcomes, the primary caregiver, usually the mother, needs to assimilate new information and then allocate sufficient time and resources to adopt and perform development promoting behaviours (such as more engaged feeding, communication, play and stimulation activities) (4, 18). However, women face many competing demands on their psychological and physical resources and often return to familiar habits and behaviours in the short- or long-term, depending on the extent of the constraints they may face (19).

Using data collected within a cluster randomised controlled trial (cRCT) of a nutrition and care for development intervention in rural India, this study explores maternal time investment in caregiving activities for children under the age of 2. Specifically, this study will explore:

(a) patterns of maternal time use to understand how time is allocated between childcare and other activities when children are aged 12 months and 18 months;
(b) whether there is a difference in maternal time use between the intervention and control arms of the cRCT of a nutrition and care for development intervention; and
(c) whether and how time allocation between childcare and other activities that mothers engage in could change as children grow older.

This may help unpack the black box of programme mechanisms that rely on behaviour change to achieve effect and reflect on intervention design to achieve maximum and sustainable impact.

## 2. Methods

### 2.1. Study setting

This study is nested within the Sustainable Programme Incorporating Nutrition and Games (SPRING) trial in India and Pakistan. Details about SPRING are presented elsewhere (20). In brief, SPRING aimed to develop and test an integrated, culturally appropriate, equitable and sustainable community-based intervention package that promotes child growth and development. SPRING involved the delivery of a home visit education and enablement package from the fifth month of pregnancy, through the first 24 months of life. The intervention design was based on formative research conducted in the study settings, where mothers reported that they felt stretched by multiple commitments and sometimes overwhelmed by their financial circumstances (21). Therefore, the intervention package was developed keeping these constraints in mind; for example, families were encouraged to support mothers so that they could engage with the intervention. SPRING was designed to be feasible, affordable and scalable through the national healthcare system.

During pregnancy, home visits made by a project-employed community-based agent (CBA) focused on maternal health, and sensitization about breastfeeding. Visits made during the post-natal period focused on breastfeeding, complementary and responsive feeding, communication, interaction and play activities. The CBAs used a counselling approach based on cognitive behaviour therapy (21), which comprised family support; guided discovery using pictures; behavioural activation; empathic listening; problem-solving; and praise. Within the child development component, CBAs explained child stimulation activities, and demonstrated these if required. CBAs also coached families on key elements such as praising children and scaffolding new activities. CBAs were trained to use counselling cards that included the instructions and key messages the CBA was to deliver in each visit, as well as culturally appropriate illustrations for the family to look at (22).

In India, SPRING was implemented in Rewari, a predominantly rural district of Haryana in North India. Haryana has a population growth rate that exceeds the national rate by approximately 0.68% (23, 24). Female literacy in the state is approximately 66%, and the sex ratio is 879 females for every 1,000 males, far below the national average of 943 females per 1,000 males (25). Haryana has among the highest rates of child mortality and undernutrition in India (23, 25).

The intervention was evaluated using a cRCT design where 12 clusters were randomly allocated to receive the intervention and 12 clusters to a control arm. Each cluster was the catchment area of a health sub-centre with a functional Auxiliary Nurse Midwife that covers a population of at least 5,000. The target population were all pregnant women and mothers with babies aged less than two years of age living within the clusters. Estimates indicate that approximately 5,000 mothers and their babies received the home visits during the duration of the programme. The primary outcomes were height-for-age, and Bayley Scales of Infant Development III, and were collected from a sub-set of the target population with children aged 18 months. Intermediate outcomes were collected from when children were aged 12 months and 18 months.

SPRING offered mothers support to create stimulating and nurturing home environments for their young children to improve their children's nutrition and development outcomes by increasing the level of investment inputs for child health and development. Data on maternal time use as a potential investment input was collected to assess whether there were differences in the time that mothers spent interacting with their children between the intervention and control arms, and how this time investment may change as children grow older.

Ethical approval for this study was granted by the London School of Hygiene & Tropical Medicine (LSHTM) Research Ethics Committee (UK), and the Sangath Institutional Review board (India). Approval was also granted by the Indian Council of Medical Research's Health Ministry Screening Committee. Informed written consent was obtained from mothers at enrolment into the trial as well as at the time of the assessments carried out when the child was aged 12 and 18 months.

### 2.2. Tool development and in-field implementation

Quantitative data on maternal time-use were collected using a survey instrument designed by the study team. The instrument was based on narrative history, a tool adapted from clinical practice (26) but not often used to collect time-use data. The instrument began by identifying the most recent normal day, which served as a frame of reference for the respondent. The most recent normal day was defined as one where there was no festival, wedding, death or funeral and no long absences from the house, i.e., where the respondent's normal routine had not been disrupted.

Once the most recent normal day had been identified, the mother was asked for their time of waking on that day. They were asked about each successive activity undertaken, asked for a brief description of the activity together with how long they spent doing that activity (primary activity), and whether they did another activity at the same time to capture multi-tasking (secondary activities). They ended by reporting the time they went to bed. The narrative history style of interviewing did not ask specific questions about doing or not doing certain types of activities. As such, it allowed mothers to recount the activities that they engaged in chronologically, reducing the risk of social desirability bias (27). This also allowed them to formulate responses rooted in their own perspective and reflecting their priorities, using the language and phrases that they felt comfortable using (28).

Before the instrument was tested in field, a pilot version was shared with the principal investigators and field coordinators to discuss the content and acceptability of the questions. The instrument was then translated into Hindi and checked for consistency with the original English version. The instrument was tested in field using cognitive interviewing techniques to establish comprehensibility, acceptability, and local relevance. The instrument was pre-tested on nine women aged between 18 and 50 years who were either pregnant or had at least one child under the age of 2 years. The women were from heterogenous socio-economic backgrounds that were representative of the site population. Cognitive interviewing was conducted by the study team in Hindi. The cognitive interviews took between 11 and 30 min to administer, with an average time of 20 min. This testing process confirmed that participants could follow all questions and that the flow of questions was acceptable.

The cognitive interviews were followed by a focus group discussion (FGD) that aimed to generate a near exhaustive list of activities that women with children under the age of 2 years might engage in on a normal day. The FGD was conducted with 12 women between 18 and 50 years of age, who were either pregnant or had at least one child under the age of 2 years and were from varied socio-economic backgrounds. We provided the discussants with the list of activities generated from the cognitive interviews as a starting point. We asked them to think about a normal day in their lives, think about what they did on that day, reflect on the activity list provided and add or remove activities. When suggesting activities, in addition to thinking about their own routine, discussants were also asked to think about what activities other women in their villages undertook. This generated 53 activities, listed by women, that we grouped into broader categories of activities that allowed for the ease of data collection and analysis, without inhibiting how respondents described the activities they engaged in. The tool and list of activities are included in Supplementary Appendix S1.

### 2.3. Data collection

Data were collected from a sub-sample of mothers when their children were aged 12 months, and again when the children were aged 18 months, to facilitate a comparison of maternal time use across different child ages, in addition to comparing time use between trial arms. Data were collected between 2016 and 2017.

We were unable to find data from the study setting or a comparable setting to inform assumptions about maternal time use for the control group. Thus, we adopted a pragmatic approach, and based the sample size of this study on the sample size calculations for an early life stress (ELS) sub-study that was nested within the SPRING trial (29). The aim of the ELS sub study was to explore the effect of adversity on growth. A minimum of 25 participants per cluster was needed to give 90% power at the 5% level of significance to detect effect sizes between 0.4SD and 0.5SD. We assumed an intra-cluster correlation of 0.05, and used an established formula (30). Our aim was to collect data from the same cohort at both data collection points. While every attempt was made to maintain the sample size at the second data collection point, the sample size was reduced to 20 per cluster due to practical considerations (Table 1).

**Table 1:** Maternal time use, sample size and data collection time points.

Assessment timing	Overall sample size	Control arm	Intervention arm
12 months of child's age	704	347	357
18 months of child's age	603	300	303

### 2.4. Analytical strategy

As Less than 5% of mothers reported multi-tasking, we present time use data for the reported primary activities only. Activities were categorised as household chores, praying, income generating activities, caring for the elderly, leisure, travelling, other activities (such as personal grooming, eating a meal, studying), caring for children aged 0–2 years, and caring for children older than 2 years.

Mothers described each activity in which they engaged during the day, starting with the time they awoke and then giving start and end times for each subsequent activity. They ended by reporting the time they went to bed, giving an estimate of the time they spent asleep at night. These times were summed together to arrive at her total “reported day”. Given common reporting phenomena such as rounding e.g., reporting time in increments of rounded hours, half hours etc., heaping e.g., the tendency to report common increments of time such as “one hour,” and the risk of recall bias, many of these reported days did not sum to 24 h. As such, to allocate each woman only 24 h of time in a day, the time spent on each individual activity and the time spent sleeping at night, was calculated as a percentage of her reported day. This percentage was then multiplied by 24 to normalise all responses to a 24-hour day.

As these data are collected within a cRCT, we tested for statistically significant differences in maternal time use between the arms using linear regression. We adjusted for potential correlation in outcomes in children within clusters by the inclusion of cluster ID as a random intercept in multilevel regression models (30). We also test for significant differences in the overall time spent on different activities when children are 12 months of age vs. 18 months of age, using linear regression with cluster as a random effect.

## 3. Results

The average age of the mothers in our sample was 23.37 years (SD 3.97, range 14–51). All mothers lived in rural areas. The average number of household members was 6.08 (SD 2.56, range 2–25). The majority of women had completed 10–12 years of schooling (36.36%), and were married and Hindu (Table 2). Similar proportions were from a general caste (34.52%) and other backward castes (36.65%). The majority of mothers lived in households with improved sources of drinking water such as household connections, public standpipes, protected dig wells and boreholes, and lived in household made from “pucca” materials such as cement, burnt bricks, concrete, timber or stone. However, the majority used unimproved cooking fuels (wood, charcoal, grass and shrubs), and had access to unimproved sources of sanitation such as a dry toilets, open spaces, and pit latrines without a flush.

**Table 2:** Sociodemographic characteristics of the study population (*n* = 704).

Socio-demographic characteristics	Frequency	Percentage
Mother's education level*		
Never been to school	34	4.83
Less than 5 years	26	3.69
5 to 9 years	180	25.57
10–12 years	256	36.36
More than 12 years	207	29.41
Marital status		
Widow	34	4.83
Divorced/seprated	1	0.14
Married	669	95.03
Religion		
Hindu	701	99.57
Muslim	2	0.28
Christian	1	0.14
Caste		
General	243	34.52
Scheduled caste	188	26.7
Other backward caste	258	36.65
Scheduled tribe	15	2.13
Source of drinking water		
Improved sources of water	657	93.32
Unimproved sources of water	47	6.68
Type of sanitation facility		
Flush/pour flush toilet	159	22.59
Pit latrine without a flush	396	56.25
Dry toilet	3	0.43
Bush, field, open space	146	20.74
Type of cooking fuel used		
Improved/clean sources	266	37.78
Unimproved sources	438	62.22
Roof material		
Kutcha materials	2	0.28
Pucca materials	699	99.29
Others	3	0.43
Wall material		
Kutcha materials	9	1.28
Pucca materials	695	98.72
Floor material		
Kutcha materials	42	5.97
Pucca materials	662	94.03

*One missing observation.

We present maternal time use in the intervention and control arms for the subsample assessed when the child was 12 months of age (Table 3) and the subsample assessed when the child was 18 months of age (Table 4).

**Table 3:** Mean number of hours spent on different activities, last normal day, 12 months.

Activity	Overall	Control	Intervention	I-C difference	*p*-value
	Mean (SD)	Mean (SD)	Mean (SD)	(95% CI)	
Total respondents	704	347	357		
Household chores	6.556	6.460	6.649	0.188	0.551
	(2.424)	(2.306)	(2.532)	(−0.466/0.843)	
Praying	0.015	0.016	0.014	−0.002	0.654
	(0.071)	(0.075)	(0.066)	(−0.012/0.008)	
Income generating activities	0.364	0.336	0.391	0.055	0.560
	(1.025)	(0.919)	(1.119)	(−0.145/0.257)	
Caring for the elderly	0.002	0.000	0.003	0.003	0.054
	(.0263)	(0.000)	(0.036)	(0.000/0.007)	
Leisure	2.970	2.950	2.990	0.040	0.771
	(1.652)	(1.601)	(1.701)	(−0.386/0.467)	
Travelling	0.212	0.179	0.244	0.064	0.358
	(0.846)	(0.813)	(0.877)	(−0.079/0.209)	
Other activities	0.864	0.908	0.821	−0.086	0.438
	(1.312)	(1.216)	(1.399)	(−0.304/0.131)	
***Child care activities (0–2 years)***	3.399	3.392	3.406	***0.013***	***0***.***990***
	(2.124)	(1.873)	(2.344)	***(−0.591/0.618)***	
Caring for children (general)	0.540	0.575	0.506	−0.068	0.396
	(0.529)	(0.555)	(0.502)	(−0.192/0.054)	
Bathing children	0.229	0.248	0.211	−0.036	0.287
	(0.459)	(0.624)	(0.191)	(−0.108/0.034)	
Feeding/breastfeeding the baby	1.859	1.803	1.913	0.109	0.611
	(1.368)	(1.155)	(1.547)	(−0.259/0.477)	
Changing baby's clothes/diaper	0.142	0.159	0.126	−0.033	0.104
	(0.168)	(0.191)	(0.140)	(−0.072/0.006)	
To calm/quieten a crying baby	0.163	0.165	0.162	−0.003	0.964
	(0.459)	(0.429)	(0.487)	(−0.115/0.109)	
Playing with children	0.462	0.439	0.485	0.045	0.596
	(0.876)	(0.782)	(0.958)	(−0.115/0.206)	
Talking or reading to the child	0.001	0.001	0.001	0.001	0.710
	(0.024)	(0.017)	(0.029)	(−0.003/0.004)	
***Caring for children older than 2 years***	0.283	0.257	0.308	***0.05***	***0***.***340***
	(0.544)	(0.505)	(0.579)	***(−0.057/0.159)***	
In bed during the night	9.331	9.292	9.370	0.077	0.704

**Table 4:** Mean number of hours spent on different activities, last normal day, 18 months.

Activity	Overall	Control	Intervention	I-C difference (95% CI)	*p*-value
	Mean (SD)	Mean (SD)	Mean (SD)		
Total respondents	603	300	303		
Household chores	6.617	6.402	6.831	0.428	0.169
	(2.479)	(2.327)	(2.607)	(−0.200/1.050)	
Praying	0.017	0.012	0.023	0.01	0.155
	(0.079)	(0.061)	(0.093)	(−0.004/0.026)	
Income generating activities	0.524	0.535	0.514	−0.021	0.898
	(1.319)	(1.373)	(1.266)	(−0.332/0.289)	
Caring for the elderly	0.011	0.002	0.018	0.015	0.207
	(0.149)	(0.030)	(0.208)	(−0.008/0.039)	
Leisure	4.545	4.818	4.274	−0.544	0.103
	(2.560)	(2.988)	(2.019)	(−1.22/0.138)	
Travelling	0.418	0.393	0.442	0.049	0.766
	(1.292)	(0.944)	(1.563)	(−0.298/0.396)	
Other activities	0.599	0.652	0.547	−0.104	0.410
	(1.028)	(1.107)	(0.942)	(−0.364/0.155)	
***Child care activities (0–2 years)***	2.595	2.687	2.503	***−0.184***	***0***.***549***
	(2.507)	(2.904)	(2.041)	***(−0.817/0.448)***	
Caring for children (general)	0.508	0.607	0.410	−0.196	0.215
	(1.512)	(2.059)	(0.581)	(−0.519/0.125)	
Bathing children	0.240	0.236	0.244	0.008	0.762
	(0.273)	(0.219)	(0.319)	(−0.046/0.062)	
Feeding/breastfeeding the baby	1.347	1.370	1.324	−0.045	0.719
	(1.414)	(1.395)	(1.433)	(−0.302/0.212)	
Changing baby's clothes/diaper	0.103	0.096	0.110	0.014	0.546
	(0.162)	(0.137)	(0.184)	(−0.033/0.061)	
To calm/quieten a crying baby	0.085	0.063	0.107	0.044	0.467
	(0.339)	(0.241)	(0.413)	(−0.075/0.164)	
Playing with children	0.293	0.292	0.294	0.002	0.978
	(0.692)	(0.650)	(0.732)	(−0.16/0.165)	
Talking or reading to the child	0.016	0.022	0.011	−0.011	0.324
	(0.137)	(0.177)	(0.079)	(−0.031/0.009)	
***Caring for children older than 2 years***	0.485	0.476	0.493	***0.017***	***0***.***890***
	(0.829)	(0.780)	(0.875)	***(−0.202/0.237)***	
In bed during the night	8.187	8.226	8.148	−0.078	0.558
	(1.209)	(1.193)	(1.227)	(−0.349/0.192)	

When the children were aged 12 months, mothers spent most of their day on household chores (over 6 h and 30 min) (Table 3). Mothers spent approximately 22 min engaging in activities that contributed to household income, and three hours on leisure activities. Approximately three and a half hours were spent on childcare activities for children under the age of 2 years. This included bathing children, feeding/breastfeeding, changing clothes/diapers, talking, or reading to children, playing with children. Of these activities, the longest duration was spent feeding/breastfeeding children (approximately 1 h and 50 min), while the shortest was on talking or reading to children. No significant differences between time-use were observed between the intervention and controls arms (Table 3).

When the children were 18 months old, continued to spend the majority of their day on household chores (Table 4). They spent approximately 31 min engaging in activities that contributed to household income, and four and a half hours on leisure activities. Approximately two and a half hours were spent on childcare activities for children under the age of 2 years. Of these activities, the longest duration was spent feeding/breastfeeding children (approximately 1 h and 20 min). No significant differences between time-use were observed between the intervention and controls arms (Table 4).

Table 5 compares the mean number of hours that mothers reported spending on different activities in the last normal day between the two time points. There were several differences between how mothers of 18-month-old children spent their time, compared with mothers of 12-month-old children. The biggest difference was seen in the time spent on leisure. Mothers with children aged 18 months spent an average of approximately 95 min more on leisure than mothers of children aged 12 months (*p*-value = 0.000). Other differences included time spent on:

• income generating activities, where mothers with older children spent approximately 10 min more on income generating activities per day (*p*-value = 0.013);
• travelling, where mothers with older children spent approximately 12 min more time travelling (*p*-value = 0.000); and
• other activities, where mothers with younger children spent approximately 16 min more time on other activities (*p*-value = 0.000)

**Table 5:** Difference in mean number of hours spent on different activities, last normal day, between 12 and 18 months.

Activity	Overall	Overall	Difference between 18 and 12 months	*p*-value
	(12 months)	(18 months)		
Total respondents	704	603		
Household chores	6.556	6.617	0.061	0.666
	(2.424)	(2.479)	(–0.317/0.439)	
Praying	0.015	0.017	0.002	0.604
	(0.071)	(0.079)	(–0.004/0.008)	
Income generating activities	0.364	0.524	0.16	0.013
	(1.025)	(1.319)	(–0.010/0.331)	
Caring for the elderly	0.001	0.010	0.008	0.135
	(0.026)	(0.149)	(–0.004/0.0213)	
Leisure	2.970	4.545	1.574	0.000
	(1.652)	(2.560)	(1.264/1.883)	
Travelling	0.212	0.418	0.206	0.000
	(0.846)	(1.292)	(0.013/0.399)	
Other activities	0.864	0.599	–0.264	0.000
	(1.312)	(1.028)	(–0.445/–0.084)	
***Child care activities (0–2 years):***	3.399	2.595	***−0.804***	***0***.***000***
	(2.124)	(2.507)	***(–1.232/–0.376)***	
Caring for children (general)	0.540	0.508	–0.032	0.632
	(0.529)	(1.512)	(–0.193/0.129)	
Bathing children	0.229	0.240	0.01	0.618
	(0.459)	(0.273)	(–0.034/0.055)	
Feeding/breastfeeding the baby	1.859	1.347	–0.511	0.000
	(1.368)	(1.414)	(–0.737/–0.286)	
Changing baby's clothes/diaper	0.142	0.103	–0.0389	0.000
	(0.168)	(0.162)	(–0.074/–0.003)	
To calm/quieten a crying baby	0.163	0.085	–0.077	0.001
	(0.459)	(0.339)	(–0.166/0.010)	
Playing with children	0.462	0.293	–0.169	0.000
	(0.876)	(0.692)	(–0.289/–0.049)	
Talking or reading to the child	0.001	0.016	0.015	0.004
	(0.024)	(0.137)	(0.004/0.025)	
***Caring for children older than 2 years***	0.283	0.485	***0.202***	0.000
	(0.544)	(0.829)	***(0.099/0.304)***	
In bed during the night	9.331	8.187	–1.144	0.000
	(1.360)	(1.209)	(–1.424/–0.865)	

There were also significant differences in time spent on childcare activities between mothers of younger and older children. Mothers spent almost 3.4 h of their day on childcare activities, when the child was 12 months of age. This reduced by 48 min when the child was 18 months of age (*p*-value = 0.000) (Table 5). Within this group of activities, the majority of time was spent on feeding/breastfeeding (approximately two hours). This sub-category remained the most time-consuming childcare activity when the child was 18 months of age but reduced by half an hour a day (*p*-value 0.000). The time spent playing with children also decreased between the two time points by approximately 10 min (*p* = 0.000). Mothers reported spending less time on changing babies’ clothes/diaper (*p*-value = 0.000) between the two time points but while significant, these time differences are very small at 2 min. Mothers of children of 12 months of age spent almost 5 min more time calming and quietening a crying baby (*p*-value = 0.001). Mothers of children of 18 months of age spent approximately 1 min more time talking or reading to the child (*p*-value = 0.004). The time spent caring for children older than 2 years increased by 12 min when the SPRING trial child was 18 months old.

## 4. Discussion

In this study, we aimed to explore maternal time use as an investment in ECD in rural India. Within the framework of the SPRING trial, we explored whether there was a difference in maternal time allocated to childcare activities and other activities, between the intervention and control arms, and how this time investment might change as children grow older.

We found that when children were 12 and 18 months old, mothers tended to spend most of their waking day on household chores. When children were aged 12 months, mothers spent an average of three and a half hours a day caring for children under the age of 2 (bathing, feeding, breastfeeding, playing, etc). Within this category of activities, the highest allocation of time was to feeding or breastfeeding children, with a lower level of time investment in playing with children and talking or reading to children. When children were aged 18 months old, mothers spent an hour less on childcare activities, although the pattern of time use withing this category of activities is similar to that seen when children were aged 12 months i.e., the highest allocation of time was to feeding or breastfeeding children, with a lower level of time investment in playing with children, and talking or reading to children. It may possibly be as that the mothers of 18-month-old children have other, younger children to whom caregiving time is allocated. These results provide us with a consistent pattern of maternal time investment in ECD.

We also found that when children were 18 months old, mothers spent more time on leisure activities and income generating activities than they did when children were 12 months old. Less time was also spent on caregiving activities for children under the age of two years with decreases in time spent feeding/breastfeeding, playing changing babies' clothes/diaper but an increase in time spent talking or reading to the child, though some of these changes were very small. There was a small increase in the time spent on caring for children older than two years.

However, no statistically significant differences were found between the intervention and control arms at either time point, indicating that the intervention did not appear have a statistically significant effect on the level of time investment for ECD. The process evaluation component of SPRING indicates that there were implementation challenges in both sites, and could account for this (31). To be able to incorporate a new activity, women would either need to stop engaging with a current activity to replace it with the new one, or reallocate their current allocation to make time for a new activity (32). This may not be possible in settings where there are constraints on women's decision-making and bargaining powers. Estimates from the 2015–2016 Indian National Family Health Survey (the year closest to the SPRING data collection) indicate that approximately 88% of currently married women (aged 15–49 years) participated in household decisions related to major purchases, their own health care and visits to family or relatives. In the state of Haryana, this was slightly lower (86%) (33). More recent estimates from the 2019–2020 Indian National Family Health Survey, indicate that women's participation in household decisions related to major purchases, their own health care and visits to family or relatives remains stable, with the proportion in Haryana being similar to that across the country (34). Both surveys found that that involvement in decision making was higher among older, wealthier and employed women, and women who lived in urban areas (33, 34). Thus, younger, and less wealthy women in rural areas are likely to have lower decision-making and bargaining power; a possible explanation for why women were not able to invest more time in ECD activities in our sample.

There also may be an opportunity cost to this time investment for households that face income constraints (32, 35). In such cases, there are likely competing priorities especially where women are expected to concurrently nurture children and their families as well as make important economic contributions (19, 32). As children grow older, it might seem that they are more independent or need less supervision, women reallocate their time to income generation activities or take time for themselves, for their own well-being (36).

Levels of parental investment have been shown to be positively correlated with parents' beliefs about the productivity of those inputs and/or the return on the level of investment (37). Thus, it is worthwhile to formally explore the role that beliefs and norms may play in ECD through the process evaluation of implemented programmes (31). This may help unpack the facilitators of or barriers to higher time investments that might arise from beliefs that mothers and other caregivers have about the effectiveness of time investments for ECD, relative to investment in more material resources such as buying toys (4, 35).

The strength of our study is that we adapted the narrative history methodology to collect time use data, which allowed women to report on the activities that they engaged in chronologically, from their own perspectives, reducing the risk of social desirability bias. This provided us with rich and detailed data. By restricting the frame of reference to be the most recent normal day, we also reduced some of the noise attributed to recall bias. However, time use data are notoriously difficult to collect because of the relative nature of time, which does have an impact on recall, especially in resource constrained settings where individuals may not formally keep time.

## 5. Conclusion

ECD programmes can have a positive impact on children's outcomes either directly as a stand-alone investment in children's development or indirectly by increasing parental investment or improving the effectiveness of existing material and time investments. Both pathways require the incorporation of new behaviours by participants, usually the primary caregiver (who tends to be the mother). We find that within a relatively short period of time in early childhood, maternal (or caregiver) time use can change, with time allocation being diverted away from childcare activities to others. This suggests that changing maternal time allocation in resource poor households may be quite challenging, and not allow the uptake of new and/or optimal behaviours. To promote effective and sustainable participation, and the uptake of ECD interventions, strategies should consider aspects related to bolstering women's decision-making and bargaining power, and support from family members such as fathers to allow women to increase their time investment, and manage available resources within their roles in households (19).

## Data Availability

The datasets presented in this article are not readily available due to patient data protection, but data are available upon reasonable request. Requests to access the datasets should be directed to n.batura@ucl.ac.uk.
