# Supplementary material for: Maternal time investment in caregiving activities to promote early childhood development: evidence from rural India
Source: Front Pediatr. 2023 Jul 7;11:1120253. doi: 10.3389/fped.2023.1120253 (PMC10360126; doi:10.3389/fped.2023.1120253)
Supplement: Supplementary file 1 [file Datasheet1.pdf]

Supplementary Material 1: Maternal time use tool, with activity list

Instruction to assessor: If there are other family members within hearing distance, ask the mother-in-law or other senior family member politely if it is possible to go to a space to ask the mother some questions alone. If this is possible, go to that private space before asking the questions. If not, consider how best to conduct these questions privately. This may mean waiting for a suitable time later in the assessment.

Explain purpose of next set of questions: To understand a woman's life, it is important to understand how women use their time. We are interested to know how you
spend your time in a normal day.

Explain what is meant by a normal day: A normal day is one where you go about your usual routine, without disruption. This means that: (1) there is no festival, wedding, any special programmes or other event such as a death in the family; (2) you did not spend a long time away from home; and (3) close family members (including yourself) were not ill.

Now I would like you to think about yesterday.

1. Was yesterday a normal day? 1. Yes 2. No<br>**[If '1', 'yes', draw a line through remainder of this table and go to 6]**	
2. Why was yesterday not a normal day?<br>**[Do not read out list. Circle most appropriate option. If mother lists more than one reason, ask her to choose the main one].**	1. Festival
	2. Wedding, other family event or visitors at home
	3. Death of a family member
	4. Illness/ accident of a family member, including yourself
	5. Visit to a nearby city, town or village; market or health centre
	6. Other (specify) ______________________________
3. Was the day before yesterday a normal day? 1. Yes 2. No<br>**[If '1', 'yes', draw a line through remainder of this table and go to 6]**	

4. Why was the day before yesterday not a normal day?<br><br>**[Do not read out list. Circle most appropriate option. If mother lists more than one reason, ask her to choose the main one].**	1. Festival<br>2. Wedding, other family event or visitors at home<br>3. Death of a family member<br>4. Illness/ accident of a family member, including yourself<br>5. Visit to a nearby city, town or village; market or health centre<br>6. Other (specify) ______________________________
5. Can you tell me what the most recent normal day was?	Description (not for entry):<br><br>Date (for entry):<br>d d - M M M - y y y y

You told me that [identified normal day] was a normal day. I would like to ask you some questions about what you did on that whole day. Please tell me about all the activities that you did that day, from when you woke up in the morning until you went to bed at night. No activity is too big or too small, I would like to know about everything that you did.

6. Firstly, at about what time did you wake up in the morning?	h h : m m

Now I'd like to ask you about what you did from that point in time onwards.

Now ask the mother about everything she did during the day yesterday. For anything to do with a child, make sure to ask the age of the child.

In column A: write down the activity

In column B: write down the time this took

In Column C: write down anything the mother did at the same time

In Column D: write down the time this took

Fill in the codes shaded in grey at the end of the interview.

Keep filling in the table until the mother *has told you about the entire day's activities. <u>At this point, draw a diagonal line through the remaining rows.</u>*

**Column A**		**Column B**	**Column C**		**Column D**
	**Code**	7b. How much time did you spend on this?	7c. Did you do anything else at the same time as this? **[If 'yes', write activity. If no, draw a diagonal line through Column C to D, and codebox. Go to next row]**	**Code**	7d. How much time did you spend on this?
7a. What was the first thing you did on waking up?		h h : m m			h h : m m
What did you do after this?		h h : m m			h h : m m
And then…?		h h : m m			h h : m m
And then…?		h h : m m			h h : m m
And then…?		h h : m m			h h : m m
And then…?		h h : m m			h h : m m
And then…?		h h : m m			h h : m m
And then…?		h h : m m			h h : m m

And then…?		hh : mm			hh : mm
And then…?		hh : mm			hh : mm
And then…?		hh : mm			hh : mm
And then…?		hh : mm			hh : mm
And then…?		hh : mm			hh : mm
And then…?		hh : mm			hh : mm
And then…?		hh : mm			hh : mm
And then…?		hh : mm			hh : mm
And then…?		hh : mm			hh : mm
And then…?		hh : mm			hh : mm
And then…?		hh : mm			hh : mm
And then…?		hh : mm			hh : mm
And then…?		hh : mm			hh : mm
And then…?		hh : mm			hh : mm

And then…?		h h : m m			h h : m m
And then…?		h h : m m			h h : m m
And then…?		h h : m m			h h : m m
And then…?		h h : m m			h h : m m
And then…?		h h : m m			h h : m m
And then…?		h h : m m			h h : m m
And then…?		h h : m m			h h : m m
And then…?		h h : m m			h h : m m
And then…?		h h : m m			h h : m m
And then…?		h h : m m			h h : m m
And then…?		h h : m m			h h : m m
And then…?		h h : m m			h h : m m
And then…?		h h : m m			h h : m m
And then…?		h h : m m			h h : m m

And then…?		h h : m m			h h : m m

8. At about what time did you go to bed?	h h : m m

Ending:

Thank you. That is the end of this set of questions.

Note to data entry operator: lines drawn through any table should be coded as '9' or '99' as appropriate.

Activity code box:

CODE	ACTIVITY	EXAMPLES
Around the home		
01	Cooking and food preparation	▫ Serving food to other family members<br>▫ Packing lunch for husbands<br>▫ Cooking a meal, including food preparation such as peeling/cutting vegetables, kneading atta etc;<br>▫ Making tea<br>▫ Preparing snacks/food for children who have returned from school<br>▫ Heating and serving milk to adults<br>▫ Heating and serving milk to children<br>▫ Making ghee or butter<br>▫ Packing lunch for school-going children
02	Cleaning the home	Cleaning/tidying up/sweeping or mopping kitchen, rooms, yard, etc
03	Washing and ironing clothes	Washing clothes and ironing them
04	Washing dishes	Washing dishes
05	Personal grooming	▫ Fresh up (go to the toilet)<br>▫ Bathing<br>▫ Dressing self<br>▫ Combing hair
06	Caring for elderly/ill household members	Feeding elderly/ill household members, helping them dress, bathe, etc
07	Eating a meal	Eating a meal, drinking beverages
08	Watching TV or listening to radio	▫ Watching TV<br>▫ Listening to the radio
Outside the home		
09	Looking after household livestock/ animals	▫ Cleaning cattle, livestock/animal sheds or enclosures<br>▫ Feeding, bathing cattle/livestock
10	Income generating activity for the household	▫ Milking cattle/ collecting eggs<br>▫ Working on the household farm or business<br>▫ Wage labour<br>▫ Working in own shop/parlour

		▫ Tailoring
11	Collecting fuel/ preparing dung cakes	▫ Making dung cakes<br>▫ Collecting firewood<br>▫ Cutting firewood
12	Collecting water	Collecting/ filling water
13	Travelling	▫ Taking/picking up children to/from school, anganwadi centre, bus stop, friend's house, etc<br>▫ Going to/ coming back from the temple/ market/ health facility
14	At the market	At the market
15	At the health centre	At the health centre
Children: Under 2 years		
16	Waking the child and putting it to bed (Under 2)	▫ Waking the child from sleep in the morning or during the rest of the day<br>▫ Putting to bed at night or during the day
17	Bathing the child (Under 2)	Bathing children
18	Changing baby's clothes/ diaper (Under 2)	Changing children's clothes/ diaper
19	Taking the child to the toilet (Under 2)	Taking children to freshen up (go to the toilet)
20	Breastfeeding the child (Under 2)	Breastfeeding children
21	Feeding the child (Under 2)	Feeding food and liquid other than breastmilk
22	Calming/quietening a crying child (Under 2)	Calming/quietening crying children
23	Helping very small children walk (Under 2)	Helping very small children walk
24	Playing with children (Under 2)	Playing with young children, with or without toys (UNDER 2)
25	Talking to children, singing to children or reading stories to them (Under 2)	Talking to children them, singing to them, telling them stories
Children: 2-5 years		
26	Caring for children aged 2 to 5	▫ Waking up children<br>▫ Putting children to bed<br>▫ Bathing children<br>▫ Taking children to freshen up (go to the toilet)

		▫ Dressing children
27	Playing with children aged 2 to 5	▫ Playing together with or without toys<br>▫ Telling stories, singing songs, reading stories or playing games
Children: Over 5 years		
28	Caring for children aged over 5	▫ Waking up children<br>▫ Putting children to bed<br>▫ Bathing children<br>▫ Taking children to freshen up (go to the toilet)<br>▫ Dressing children<br>▫ Helping school-going children with homework
29	Playing with children aged over 5	▫ Playing together with or without toys<br>▫ Telling stories, singing songs, reading stories or playing games
Other (either inside or outside the home)		
30	Rest	Rest
31	Sleeping	Sleeping
32	Prayer	▫ Praying at place of worship<br>▫ Praying at home<br>▫ Offering water to Sun<br>▫ Lighting incense/*diyas*<br>▫ Keeping/breaking fasts
33	Talking with friends/ family	▫ Chatting with neighbours or family members<br>▫ Talking on the phone
34	Studying	Studying
35	Exercise	Exercise
36	Other	Anything not covered by the codes
37	Heating water for other family members	Heating water for other family members for bath etc.
